# Co_x_Fe_y_@C Composites with Tunable Atomic Ratios for Excellent Electromagnetic Absorption Properties

**DOI:** 10.1038/srep18249

**Published:** 2015-12-11

**Authors:** Hualiang Lv, Guangbin Ji, Haiqian Zhang, Meng Li, Zhongzheng Zuo, Yue Zhao, Baoshan Zhang, Dongming Tang, Youwei Du

**Affiliations:** 1 College of Material Science and Technology, Nanjing University of Aeronautics and Astronautics, Nanjing 210016, P. R. China; 2 School of Electronic Science and Engineering, Nanjing University, Nanjing 210093, P. R. China; 3 Laboratory of Solid State Microstructures, Nanjing University, Nanjing 210093, P. R. China

## Abstract

The shell on the nano-magnetic absorber can prevent oxidation, which is very important for its practical utilization. Generally, the nonmagnetic shell will decrease the integral magnetic loss and thus weaken the electromagnetic absorption. However, maintaining the original absorption properties of the magnetic core is a major challenge. Here, we designed novel and facile Co_x_Fe_y_@C composites by reducing Co_x_Fe_3−x_O_4_@phenolic resin (x = 1, 0.5 and 0.25). High saturation magnetization value (Ms) of Co_x_Fe_y_ particle, as a core, shows the interesting magnetic loss ability. Meanwhile, the carbon shell may increase the integral dielectric loss. The resulting composite shows excellent electromagnetic absorption properties. For example, at a coating thickness of 2 mm, the RL_min_ value can reach to −23 dB with an effective frequency range of 7 GHz (11–18 GHz). The mechanisms of the improved microwave absorption properties are discussed.

Solving the oxidization problem of magnetic metal has aroused extreme attention in the field of electromagnetic absorption. It is well known that an ideal electromagnetic absorber should have a high impedance matching (more electromagnetic waves can be incident on the absorber with less reflection) and strong electromagnetic wave attenuation^1^,^2^,^3^,^4^. Of all the absorbers, magnetic materials have aroused more attention than pure dielectric absorbers because they have clear impedance matching properties and a desired magnetic loss ability^5^. Thus, many works related to magnetic materials have been widely studied in recent years. Currently, coin-like iron with a minimum reflection loss of −53 dB was reported by our group^6^ and the sphere-like Co_x_Fe_3−x_O_4_ with an optimal reflection loss value of −41.098 dB^7^ has been produced by Ji *et al.* Meanwhile, Tong and coworkers reported a flower-like Co with a RL_min_ value of −40.25 dB^8^. Nevertheless, despite the fact that these magnetic absorbers have achieved fascinating electromagnetic absorption properties, most magnetic metals (*i.e.*: Fe, Co, Ni) are restricted by the poor chemical stability and easily convert into nonmagnetic oxide, *i.e.* α-Fe_2_O_3_, Co_2_O_3_, etc. This process will seriously affect their magnetic properties and magnetic loss ability. Moreover, many stable ferrites such as CoFe_2_O_4_ have poor absorption properties due to their low Ms (usually below 100 emu/g). It should be explained that a high Ms is beneficial to impedance matching behavior and permeability values including the real part (*μ*′) and the imaginary part (*μ*″)^9^,^10^. The big *μ*′ and *μ*″ values are thought to be the main reason why the magnetic materials are superior to the dielectric materials (*i.e.*: ZnO, CuS, TiO_2_) according to the following equations^11^:


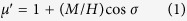



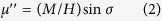


Here, *M* represents magnetization, *H* is the external magnetic field, and *σ* stands for the phase lag angle of magnetization behind external magnetic field. From equations (1, 2), we conclude that a high magnetization value is quite important. To solve the oxidation problem, a core-shell structure may be an effective strategy. For example, Yang *et al.* utilized the SiO_2_ shell to protect the cube-like Fe out of air^12^. Similarly, the SiO_2_ shell has been widely used in other types of absorbers including Ni and Fe_3_O_4_^13^,^14^. Unfortunately, these results revealed that an insulated SiO_2_ coating shell is harmful to the integral *μ*′, *μ*″ as well as to dielectric. Thus, other efforts have focused on replacing SiO_2_ with improved high dielectric loss materials.

Most dielectric materials (*e.g.* TiO_2_ and ZnO) are form shells and show high chemical stability due to their matched lattice and surface free energy^15^. Next, the high dielectric loss shell can make up a slight decrease in the magnetic loss. Among these absorbers, Fe_3_O_4_-based core-structures have been widely studied including Fe_3_O_4_@ZrO_2_^16^, Fe_3_O_4_@TiO_2_^17^, Fe_3_O_4_@SnO_2_^18^, and Fe_3_O_4_@CuSiO_3_^19^. Of course, other magnetic/dielectric structures have also been reported including Fe@SnO_2_^20^ and Co@ZnO^21^. In fact, these pristine magnetic cores did not show the interesting complex permeability value due to their smaller *Ms*. After introducing the non-magnetic material, the integral magnetic loss ability will further decrease. Therefore, a high *Ms* magnetic core is needed. Of the magnetic materials, FeCo shows a higher *Ms* than most magnetic materials. In our previous work, we successfully synthesized the hexagonal cone-like Fe_50_Co_50_ with a high *Ms* value of 225 emu/g—higher than the theoretical value of 218 emu/g. Surprisingly, the corresponding *μ*′ of Fe_50_Co_50_ is more than 2 at the early frequency region, which is larger than most of magnetic materials (usually at 1.5). Furthermore, the Fe_50_Co_50_ displays better air-stability at room temperature than Fe or Co single state^22^.

In this study, the Co_x_Fe_y_@C composite was obtained easily by using Co_x_Fe_3−x_O_4_ as the core. The carbon shell with tunable thickness in the final composite has the following advantages versus other dielectric materials. 1) Most dielectric shells cannot exist in the acid or alkaline environment while carbon can endure such unfriendly conditions. 2) The higher dielectric loss ability makes the composite exhibit multiple-attenuation ability. 3) The lower density of the carbon shell may lead to a light-weight absorber. 4) The electromagnetic absorption properties can be easily tuned by adjusting the carbon shell thickness. Such a composite offers high stability and strong absorption performance. In additional, the high Ms of Co_x_Fe_y_ may result in the magnetic loss. Furthermore, the Co and Fe atomic ratio can be tuned by adjusting the x value in the Co_x_Fe_3−x_O_4_.

## Results

**Figure 1:**
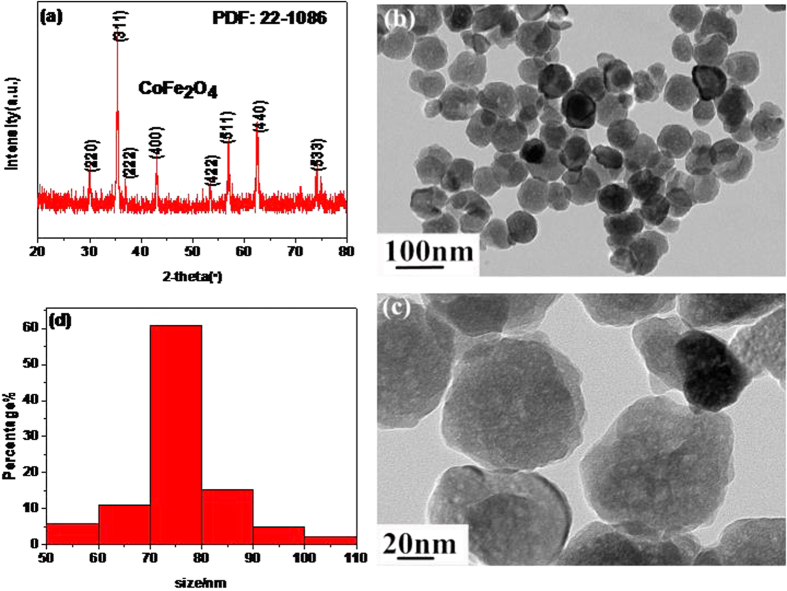
The XRD patterns (a) TEM images (b,c) and size distribution (d) of the pure CoFe_2_O_4_.

Detailed information about the representative Co_x_Fe_3−x_O_4_ (CoFe_2_O_4_ nanospheres) is listed in Fig. 1a–d. From Fig. 1a, all diffraction peaks can be matched well with the CoFe_2_O_4_ planes (PDF card No.: 22–1086). At the same time, the nanoscale CoFe_2_O_4_ presents a 60–80 nm sphere (Fig. 1b–d).

**Figure 2:**
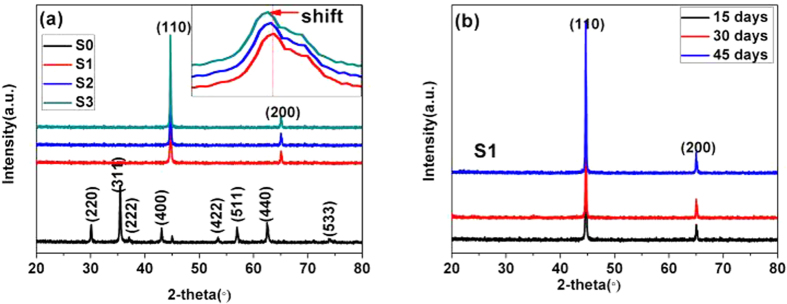
The XRD patterns of S0-S1 samples (a) and the XRD dates of the S1 tested at different days (b).

The XRD patterns of these samples are shown in Fig. 2. We infer that the carbon shell does not influence the crystal structure of CoFe_2_O_4_. That is, the CoFe_2_O_4_@C and CoFe_2_O_4_ share identical diffraction peaks. As for the Co_x_Fe_y_@C composite, the obvious diffraction peaks at 44.6 and 64.9° belong to the iron-cobalt phase. No any other impurity peaks including Fe or Co oxidation peaks can be observed indicating the high-purity of these samples. The inset reveals that the main diffraction peaks exhibit a slight left shift from S1 to S3, which is consistent with Aguirre’s report^23^. To demonstrate the anti-oxidation of Co_x_Fe_y_@C, sample S1 has been further characterized on different days (Fig. 2b). Obviously, there is little change after S1 is exposed to air for 15, 30, and 45 days. The atomic ratios of each sample tested by ICP (Co/Fe ratio: S1: 1/1.97; S2: 1/5.01; and S3: 1/11.05) are close to the initial Fe^3+^ and Co^2+^ ratio (S1: CoFe_2_; S2: CoFe_5_; and S3: CoFe_11_).

**Figure 3:**
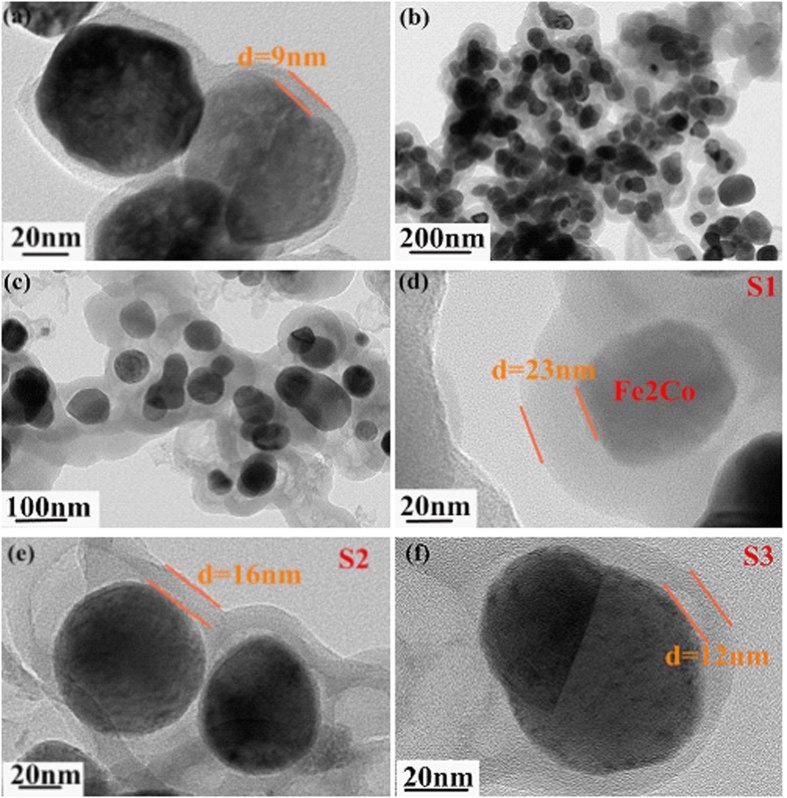
The TEM images of the S0 (a), S1 (b–d), S2 (e) and S2 (f).

The detailed core-shell structures of these products were investigated by TEM. In Fig. 3a, the carbon shell with a shell thickness of 9 nm is obvious. As described in Fig. 3b, CoFe_2_ was surrounded by a clear carbon layer. The thickness of the carbon shell is up to 23 nm (see Fig. 3d). Meanwhile, it is worth noting that the size of the S1 is slightly decreased versus the original CoFe_2_O_4_. Such a change may be attributed to the loss of the O element and the structure shrinkage during reduction. As for S2 and S3, the corresponding carbon shell thicknesses are 16 and 12 nm, respectively.

**Figure 4:**
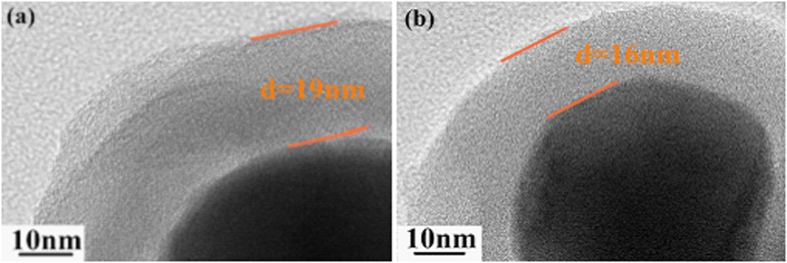
The TEM images of S1 prepared with different resorcinol mass (a) 0.25 g (b) 0.1 g.

Actually, the thickness of the carbon is tunable by adjusting the mass of resorcinol as seen in Fig. 4a,b. When the resorcinol dosage reduces to 0.25 and 0.1 g, the carbon shell thickness of S1 decreases to 19 and 16 nm, respectively (See Fig. 4).

**Figure 5:**
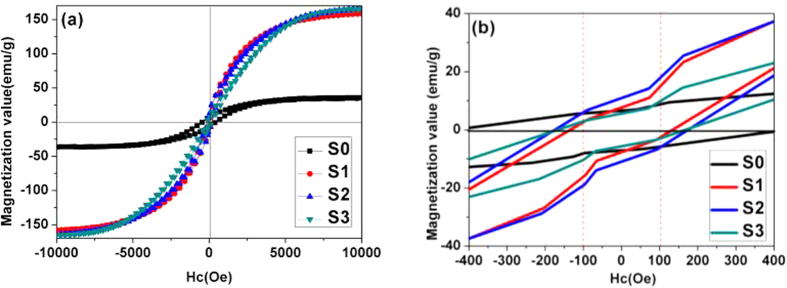
The M-H loops (a) −10 kOe-10 kOe (b) −400 Oe-400 Oe.

Figure 5 compares the magnetization loops of the composites. Generally, the Ms value of pure CoFe_2_O_4_ is below 80 emu/g^24^. When CoFe_2_O_4_ was coated with nonmagnetic carbon, the magnetization values decrease abruptly. Here, the magnetization value of S0 is only 37 emu/g, which dampens its magnetic loss ability. However, the magnetization values of S1-S3 still remain at high (160~170 emu/g), close to the Ms value of bulk Co (162 emu/g). The larger magnetization value is not only beneficial for impedance matching, but also results in strong magnetic loss ability. It is also known that a high coercive force value (*Hc*) will make the resonance peak shift to a high frequency region. Figure 5b confirms that all the *Hc* values of Co_x_Fe_y_@C composites are bigger than 100 Oe, and S3 has the highest *Hc* value of 175 Oe. The other two samples are 134 and 167 Oe for S1 and S2, respectively.

**Figure 6:**
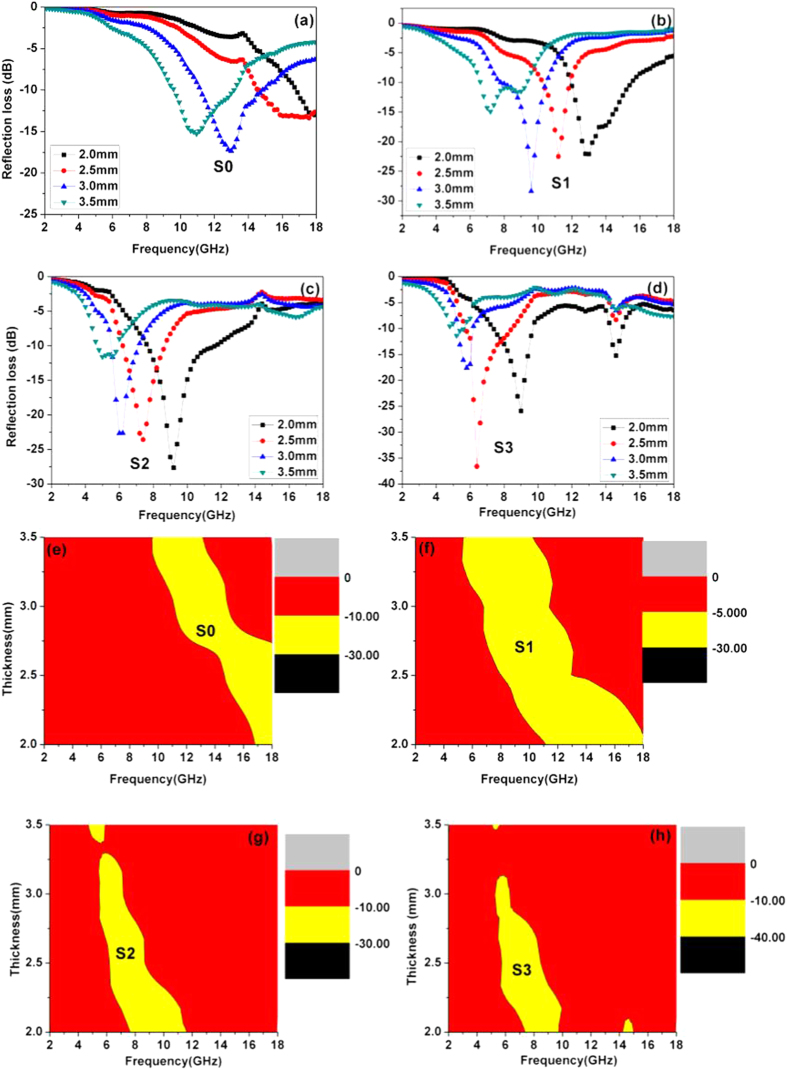
The reflection loss data (a–d) and effective frequency region (e–h) of S0–S3.

Figure 6 describes the relationship between the RL data and frequency of these samples. As seen in Fig. 6a–d, the absorption peaks of each sample move to the lower frequency region as the thickness increases. This can be explained by the 1/4 wavelength equation^25^.


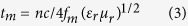


Here, *t*_*m*_ and *f*_*m*_ are the matching thickness and frequency of the RL_min_ peaks, and *c* is the velocity of light. With the carbon modification, all samples enhance the electromagnetic absorption properties versus pure CoFe_2_O_4_ (See Fig. S1). However, the Co_x_Fe_y_@C composites (S1~S3) offer an interesting RL_min_ value and effective frequency width at all the tested frequency. For example, it is clear that in Fig. 6, the optimal RL_min_ value of S0 is no more than −20 dB at a larger coating layer of 3 mm (RL_min_<−10 dB means 90% of attenuation). But, the optimal RL_min_ value for S3 is close to −38 dB with a relatively thin coating layer of 2.5 mm. The optimal RL_min_ values of S1 and S2 are up to −28 and −27 dB with a thickness of 3.0 and 2.0 mm, respectively.

The effective frequency range for an ideal electromagnetic absorber (RL_min_ < −10 dB) is another important factor to evaluate in the performance of the absorber (Fig. 6 e-h). The yellow area is indexed to the effective frequency width. It is apparent that the yellow area of the S1-S3 samples is much broader than that of the S0. Under a thin coating thickness of 2 mm, the frequency width of the S1-S3 samples is larger than 2 GHz. In particular, the frequency width of S1 is up to 7 GHz (from 11 to 18 GHz) while the S0 is no more than 1 GHz (17–18 GHz). In addition, the as-prepared S1 composite shows a superior absorption property among other similar composites as illustrated in Table 1^26^,^27^,^28^,^29^,^30^.

## Discussion

**Figure 7:**
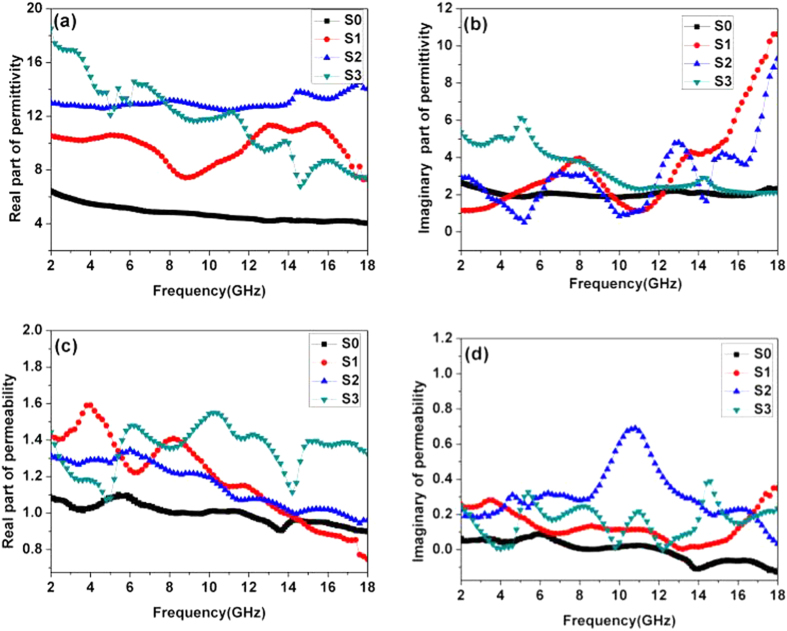
The electromagnetic parameters of S0-S3: real part (a)/imaginary part (b) of permittivity and real part (c) and imaginary part (d) of permeability.

In Fig. 7, the relevant electromagnetic parameters explain the enhancement of the electromagnetic absorption properties. When these magnetic cores are coated with the high dielectric carbon, *ε*′ and *ε*″ increase but to different extents. Within the tested frequency range, the *ε*′ value of the Co_x_Fe_y_@C samples are quite a bit bigger than that of S0 (6.2-4). This implies the increasing energy storage ability. The corresponding *ε*′ values are ranged from 11.5-7, 14-13, and 19.5-7.4 for S1, S2, and S3, respectively. It is generally believed that *ε*″ is related to its dielectric ability. As shown in Fig. 7b, these Co_x_Fe_y_@C composites achieve an ideal *ε*″ value across most of the frequency spectrum. The obvious resonance peaks are benefit the high *ε*″ values. Because of this, the *ε*″ values of the S1-S3 samples span a wide range. For instance, 1–10.5 for S1, 0.3–9 for S2 and 2–5 for S3—the S0 sample has a relatively narrow region of 2–2.8 (Fig. S2). In this framework, the larger *ε*″ of S1-S3 mainly come from the high dielectric carbon shell.

It is widely believed that pure carbon has excellent electric conduction properties. As a result, the carbon shell will increase the *ε*″ value after being composited with materials. Meanwhile, the metallic feature of the Co_x_Fe_y_ alloy core will strengthen the electric conduction and therefore further increase its *ε*″ value. In addition, the presence of resonance peaks among the S1~S3 samples also favor the intensity *ε*″ value.

Many factors may cause these resonance peaks, including ionic, electronic, atomic, interface and dipole relaxation polarization^31^. Whereas at high frequency regions, these resonance peaks are impossible to separate from electronic and atomic polarization (below GHz)^32^. Therefore, they probably result from the interface and dipole polarization. The presence interface between the Co_x_Fe_y_ core and carbon shell is apt to induce interface polarization according to similar literature^33^,^34^. At the same time, the loss of O during the reduction process may generate many lattice defects. These lattice defects act as polarization centers that are reflected in increasing *ε*′ values. Meanwhile, these dipoles will alter their direction under an external electromagnetic field. This procedure increases the attenuation of the electromagnetic energy ability^35^. At the same time, the carbon shell and Fe_x_Co_y_ shows different electrical conductivity and polarity properties. Under the external magnetic field, the composite will lead to strong interface polarization effects. Figure 7c,d reveal the real/imaginary values of permeability (*μ*′/*μ*″). The higher magnetization values of the Co_x_Fe_y_@C composites are significantly better at magnetic loss. The *μ*′ value of the S1 is up to 1.6-0.9, 1.3-1.0 for S2 and 1.6-1.1 for S3—values that are much larger than S0 (1.1-0.9). Meanwhile, the Co_x_Fe_y_@C composites share better magnetic loss advantages with all the *μ*″ values of Co_x_Fe_y_@C samples located at 0.7-0.1 and quite bigger than S0 (below 0.1) and other types of pure magnetic materials including hollow cobalt (~0.2)^36^, nanoring-like Fe_3_O_4_ (0-0.4)^37^, and hexagonal flake Fe (0.15-0.3)^38^.

In addition, there are at least two resonance peaks in these composites. These peaks are susceptible to magnetic loss due to eddy current effects, natural resonance and exchange resonance^39^. While eddy current effects can attenuate a small part of incidence electromagnetic wave, it also generates eddy current and hinders electromagnetic waves entering the absorber. The eddy current can be expressed as^40^:


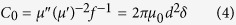


**Figure 8:**
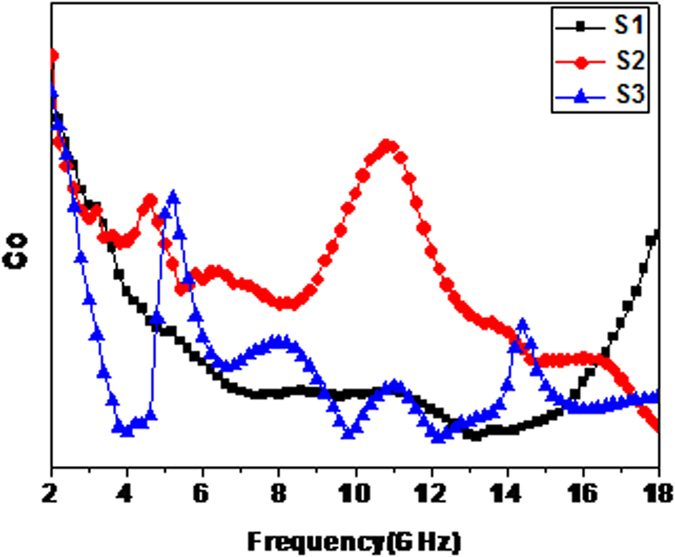
The C_0_-f curve of S1–S3.

If the resonance peak comes from the eddy current, C_0_ will be a constant. As seen from Fig. 8, these values of C_0_ change with increasing frequency and thus can rule out the eddy current. For Co_x_Fe_y_@C nanocomposites, the natural resonance is always weak and can be neglected. The small size (less than 100 nm) can arouse strong exchange resonance. In particular, the lower magnetic anisotropy may increase the magnetic moment coupling and lead to obvious exchange resonance. Thus, we can deduce that both peaks are exchange resonance.

The enhanced electromagnetic absorption property is directly controlled by the impedance matching ratio and attenuation constant α. This means that the integrated attenuation effect of magnetic and dielectric loss is based on the following equation^41^,^42^:


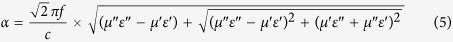


**Figure 9:**
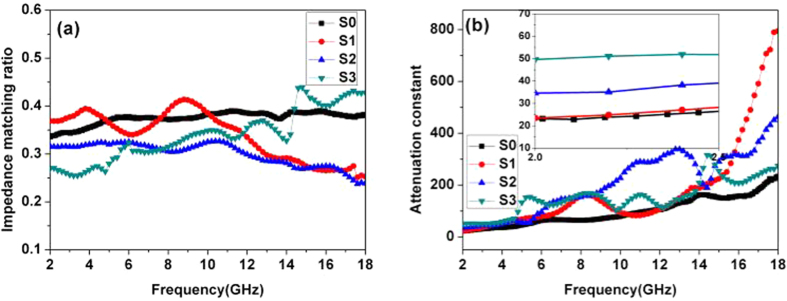
The impedance matching (a) and attenuation constant (b) of S0–S3.

Versus S0, the larger *ε*′ and *ε*″ values of S1-S3 are not favorable for impedance matching. Due to the contribution of magnetization properties, the impedance matching ratio values of S1-S3 are smaller than S0, Nevertheless, the Co_x_Fey@C composites present fascinating attenuation ability according to Fig. 9a. Figure 9b confirms all the tested frequencies, and the attenuation values of S1-S0 are much bigger than S0.

Based on this discussion, we conclude that the enhanced electromagnetic absorption mechanism can be ascribed to the following aspects. First, the high *Ms* of Co_x_Fe_y_ core can retain high magnetization values in the composite and further improve the impedance matching. Meanwhile, the obvious magnetic loss also raises the electromagnetic wave loss ability that originates from its high magnetization. Nevertheless, the magnetization value of S0 decreases to a lower value (less than 40 emu/g) after the carbon coating. Such a lower magnetization value make it weak at magnetic loss in each *μ*′ and *μ*″ value. Second, ultra-small size of the Co_x_Fe_y_ alloy is apt to form an obvious exchange resonance that effectively suppresses current eddy effects. These multi-resonance peaks also play a vital role on the RL_min_ value and frequency width. Third, due to the high dielectric loss carbon shell, the presence interface between Co_x_Fe_y_ and carbon may lead to remarkable interface polarization. The presence of lattice defects resulted from the loss of O that cause dipole polarization. The appearance of multi-resonance in the imaginary part is very favorable for electromagnetic wave loss. However, for CoFe_2_O_4_@C, we can hardly observe any obvious resonance peaks. This can be explained in that CoFe_2_O_4_ is the spinel structure and Co^2+^ occupies an A site while Fe^3+^ remains in the B site. The electron will transfer from the A to the B and increase polarization^43^. This type of polarization is weak and occurs in the interior of a single spindle structure. As a result, the CoFe_2_O_4_ core is not sensitive to the carbon shell and thus impairs the interface polarization.

## Conclusions

In summary, we took advantage of the high chemical stability and dielectric loss feature of carbon to coat magnetic metals and prevent oxidation. In this context, the high *Ms* of Co_x_Fe_y_ (CoFe_2_, CoFe_5_, and CoFe_11_) as the core increases the integral magnetic loss ability. The electromagnetic absorption between Co_x_Fe_y_@C and CoFe_2_O_4_@C demonstrates that the Co_x_Fe_y_@C is superior in microwave absorption at the RL_min_ with effective frequency. The enhanced microwave absorption can be attributed to the exchange resonance, interface and dipole polarization. The as-prepared sample has excellent stability after long-term air exposure.

## Method

Ammonium hydroxide (NH_4_OH), glucose, formaldehyde, ferric chloride (FeCl_3_), cobalt acetate (Co(Ac)_2_), urea and ethylene glycol (EG), ethanol and resorcinol were purchased from the Sinopharm Chemical Reagent Co. All chemical regents were analytically pure and used without further purification.

### Co_x_Fe_3−x_O_4_ sphere-like nanoparticles preparation

The Co_x_Fe_3−x_O_4_ sphere-like nanoparticles were synthesized by a simple solvthermal approach. The Co(Ac)_2_ and FeCl_3_ were mixed into a 30 mL EG solution for magnetic string 30 min. Then, before the mixed solution was transferred to 50 mL autoclave, 15 mmol urea was added into the solution. The autoclave was heated at 200 ^o^C for 12 h. After the temperature cooled to room temperature, the precipitate was collected by magnetic separation. The representative CoFe_2_O_4_ was prepared when x was set to 1.

### Synthesis of Co_x_Fe_y_@C

Initially, the 0.8 g of as-prepared Co_x_Fe_3−x_O_4_ (X was set as 1, 0.5 and 0.25 and marked as S1, S2, and S3) were dispersed into a solution that contains 80 mL distilled water, 20 mL ethanol solution and 1 mL NH_3_.H_2_O with ultrasonic mixing for 1 h. Next, 0.5 g resorcinol and 3 mL formaldehyde were added to the above mixture for polymerization for 1 day. The generated Co_x_Fe_3−x_O_4_@phenolic resin was reduced by hydrogen gas (V_H2_/V_N2_ = 10:90) at 500 °C for 2 h. The heating ramp ratio was controlled at 1 °C/min. For comparison, the most representative CoFe_2_O_4_@C (named as S0) was obtained in N_2_ atmosphere only and identical conditions.

### Characterization

Phase analysis was conducted depending on the powder X-ray diffraction (XRD) patterns (Bruker D8 ADVANCE X-ray diffractometer) with Cu Kα radiation (λ = 0.154178 nm with 40 kV scanning voltage, 40 mA scanning current and scanning range from 20 to 80°). The core-shell features of these composites were detected with transmission electron microscope (TEM, JEM JEOL 2100). The magnetic properties of coercive force (Hc) and magnetization date were acquired by a vibrating sample magnetometer (VSM, Lakeshore, Model 7400 series) at room temperature (298 K). The atomic ratio of Fe and Co was measured with inductively coupled plasma (ICP, Optimal 5300DV).

### Electromagnetic parameters tests

The S parameters including S11, S12, S21, and S22 were measured with an Agilent PNA N5224A vector network analyzer using the coaxial-line method. The samples were prepared by homogeneously mixing the paraffin wax and sample (mass ratio: 50:50) and then pressing into toroidal-shaped samples (Φout:7.0 mm, Φin:3.04 mm). Subsequently, the Agilent PNA software can process the *ε*′, *ε*″, *μ*′, *μ*″ values. Finally, the RL value with 2–3.5 mm can be determined by the following formulas^44^,^45^.


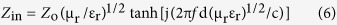



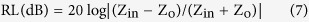


Here, *Z*_*in*_ is the input impedance of the absorber, *f* is the frequency of the electromagnetic wave, *d* is the coating thickness of the absorber and *c* is the velocity of electromagnetic wave in free space. Terms *ε*_*r*_ (*ε*_*r*_* *=* ε*′-*jε*″) and *μ*_*r*_ (*μ*_*r*_ = *μ*′-*jμ*″) are the complex permittivity and permeability of the absorber.

## Additional Information

**How to cite this article**: Lv, H. *et al.* Co_x_Fe_y_@C Composites with Tunable Atomic Ratios for Excellent Electromagnetic Absorption Properties. *Sci. Rep.* **5**, 18249; doi: 10.1038/srep18249 (2015).

## Supplementary Material

Supplementary Information

## Figures and Tables

**Table 1:** The comparison of the RL_min_ and effective frequency of other reported magnetic@dielectric composites.

Sample	Thickness	RL_min_	Effective Frequency	Ref.
FeNi@C	2.0 mm	−33.0 dB	<1.0 GHz	26
Fe_3_O_4_@C	2.0 mm	−20.7 dB	∼4.8 GHz	27
Fe@C	2.7 mm	−25.0 dB	<3.0 GHz	28
Ni@C	2.0 mm	−32.0 dB	∼4.3 GHz	29
Fe_55_Co_45_@C	2.5 mm	−15.8 dB	∼2.0 GHz	30
Fe_60_Co_40_@C	2.5 mm	−16.4 dB	∼5.0 GHz	30
This study	2.0 mm	−30.0 dB	∼7.0 GHz	
