# Supplementary material for: CoxFey@C Composites with Tunable Atomic Ratios for Excellent Electromagnetic Absorption Properties
Source: Sci Rep. 2015 Dec 11;5:18249. doi: 10.1038/srep18249 (PMC4676003; doi:10.1038/srep18249)
Supplement: Supplementary Information [file srep18249-s1.doc]

**Electronic Supplementary Information**

**Co_x_Fe_y_@C Composites with Tunable Atomic Ratios for Excellent Electromagnetic Absorption Properties**

Hualiang Lv^1^, Guangbin Ji^1,*^, Haiqian Zhang^1^, Meng Li^1^, Zhongzheng Zuo^1^, Yue Zhao^1^, Baoshan Zhang^2^, Dongming Tang^2^ and Youwei Du^3^

*^1^College of Material Science and Technology, Nanjing University of Aeronautics and Astronautics, Nanjing 210016, P. R. China*

*^2^School of Electronic Science and Engineering, Nanjing University, Nanjing 210093, P. R. China*

*^3^Laboratory of Solid State Microstructures, Nanjing University, Nanjing 210093, P. R. China.*

**Fig S1 The reflection loss date of the pure CoFe_2_O_4_**

**Fig S2 The electromagnetic parameters of pure CoFe_2_O_4_**
